# The First ongoing Pregnancy Following Comprehensive Aneuploidy Assessment Using a Combined Blastocenetesis, Cell Free DNA and Trophectoderm Biopsy Strategy

**Published:** 2019

**Authors:** Jara Ben-Nagi, Rabi Odia, Xavier Viñals Gonzalez, Carleen Heath, Dhruti Babariya, Sioban SenGupta, Paul Serhal, Dagan Wells

**Affiliations:** 1- Clinical Department, The Centre for Reproductive and Genetic Health, London, UK; 2- Embryology Department, The Centre for Reproductive and Genetic Health, London, UK; 3- Cooper Genomics, London, UK; 4- Institute of Women’s Health, University College London, London, UK; 5- Department of Women’s and Reproductive Health, University of Oxford, Oxford, UK

**Keywords:** Biopsy, Blastocentesis, Cell free DNA

## Abstract

**Background:**

The exact origin of cell-free DNA found in spent culture media or blastocoel fluid is currently unknown but with the potential to become an improved source of DNA for chromosomal analysis than trophectoderm biopsy samples, it provides a superior representation of the fetal genetic status. However, the genetic material contained within the blastocoel cavity may be more reliable to assessment of embryo euploidy in a clinical context than trophectoderm of cell-free DNA.

**Case Presentation:**

This is the first UK case report where all three sources of DNA were analyzed in a clinical setting on 29 th January 2018 at the Centre for Reproductive and Genetic Health, London, leading to an ongoing clinical pregnancy.

**Conclusion:**

The experience from this case report suggests that removal of blastocoel fluid, sampling of spent culture media and trophectoderm biopsy can be carried out in parallel. Gathering genetic information from two to three independent samples of embryo DNA may provide enhanced diagnostic accuracy and may clarify cytogenetic status of mosaic embryos.

## Introduction

Preimplantation genetic testing for aneuploidies (PGT-A) is a method that aims to describe the chromosomal complement of embryos. The use of PGT-A to identify euploid embryos, which can then be prioritized for transfer to the uterus, has been the subject of significant debate and controversy. Recent literature suggests that IVF success (Focusing on ongoing pregnancy rates per transfer) can be increased by up to a 30% when PGT-A is employed to assist embryo selection in single embryo transfer cycles (1). Other randomized controlled trials have shown significantly higher implantation and delivery rate and lower multiple pregnancy rates (2, 3). Meta-analyses and systematic reviews have reported increased ongoing pregnancy and live birth rate and decreased miscarriage rate in cycles utilizing PGT-A (4, 5). Nevertheless, some have continued to voice concerns over the clinical value of PGT-A, questioning its benefit in terms of live birth rates and expressing worries that errors in assigning embryo chromosomal status may cause some potentially viable embryos to be discarded (6, 7). Currently, embryo biopsy is an invasive but indispensable component of the PGT-A procedure. It has been argued that it would be advantageous if biopsy could be eliminated, since it entails a significant additional cost and risks placing the embryo under stress (8). Several attempts to determine whether or not an embryo is aneuploid by inference from its morphokinetic behavior have been attempted but have failed to generate sufficiently robust data to support the use of morphological and/or time-lapse systems for the determination of embryo ploidy status (9, 10). Other potential approaches for the cytogenetic evaluation of embryos, avoiding the use of embryo biopsy, have been reported in the literature. Minimally invasive or non-invasive strategies include the analysis of blastocoel fluid and spent culture media, both of which have demonstrated the presence of genetic material (11–14). However, low amplification yield and poor DNA integrity, particularly affecting blastocoel fluid, posea challenge for chromosomal analysis. Literature on blastocoel fluid describes a ploidy concordance between trophectoderm biopsy and the blastocoel fluid DNA ranging from 60 to 97% (15, 16). In the case of spent embryo culture media, concordance rates between trophectoderm biopsy and media samples reported by different groups have varied from 70 to 90% (17, 18). However, DNA contamination may be a potential problem. While most groups have not recorded an appreciable incidence of contamination, Vera-Rodriguez et al. (2018) reported high levels of maternal DNA contaminants following amplification and analysis of a polymorphic short tandem repeat (STR), with only ∼8% of the DNA fraction in the embryonic culture media being embryonic in origin. Herein, a case with the confirmation of ploidy status was reported with the aforementioned procedures and trophectoderm biopsy. This case provides direct evidence that multiple invasive and non-invasive techniques can be combined and deployed in the clinic in tandem, potentially enhancing diagnostic accuracy rates. To the best of our knowledge, this case represents the first clinical pregnancy following assessment of an embryo using all three of the aforementioned procedures.

## Case Presentation

A 43-year-old woman presented for evaluation of infertility on 29 th January 2018 at the Centre for Reproductive and Genetic Health, London. She had secondary infertility for one year and had previously had a first trimester miscarriage, which was managed conservatively. Her medical history was unremarkable. Her ovarian reserve test showed a total antral follicle count of 6, anti-Mullerian hormone 4.7 *pmol/l*, follicle stimulating hormone 10.10 *IU/l* and oestradiol <250 *pmol/l*. Her male partner was 33 years old. His medical history was unremarkable and his semen analysis was normal. The couple was counseled. The patient underwent two consecutive cycles of controled ovarian stimulation to optimize the yield of embryos sent for PGT-A testing. An antagonist protocol was prescribed with Fostimon® 225 *IU* and Merional® 225 IU (IBSA, Italy). Vaginal egg retrieval was carried out 37 *hr* post 10,000 *IU* of Pregnyl (MSD, UK).

On the first cycle, a total of eight oocytes were collected, seven were mature and three exhibited normal fertilization at 16–18 *hr* post intracytoplasmic sperm injection (ICSI). A total of three top-quality embryos were frozen using the vitrification techniques on day 3 of embryo development. Embryo vitrification was performed using Kitazato vitrification kit with the use of high concentrations of cryoprotectants and ultra-rapid cooling to avoid detrimental ice crystal formation (20). On a subsequent cycle, a total of four eggs were collected all of which were mature and three fertilized following ICSI. On day 3 of embryo development, the frozen embryos from the previous cycle were thawed and all cultured to the blastocyst stage simultaneously. From the frozen thawed cohort of embryos, one embryo reached the blastocyst stage on day 6, while from then fresh cohort one embryo formed a blastocyst on day 5 of development. The blastocyst is an advanced stage of embryo development where the embryo differentiates into two cell lines, the inner cell mass and the trophectoderm, and forms a fluid filled cavity (Blastocoel). It is a prerequisite to culture embryos to the blastocyst stage prior to trophectoderm biopsy.

**Figure 1.**
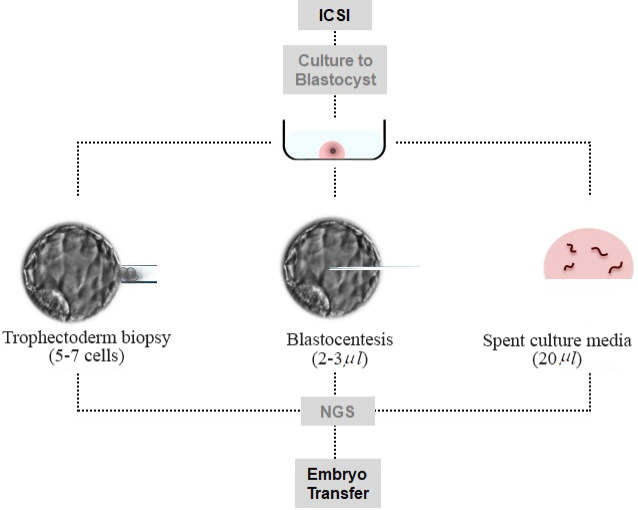
All DNA source for PGT-A were individually expelled into eppendorf tubes and stored at −80°C until analysis

The unit policy is to only perform blastocentesis on half the embryos reaching the blastocyst stage for quality control. Therefore, the day 5 developing blastocyst was subjected to blastocentesis using a microinjection pipette prior to trophectoderm biopsy (Figure 1). A minimal invasive micro-puncture was performed between two junctions of cells, the fluid was aspirated and then expelled into a 0.2 *ml* Eppendorf tube. The tube was immediately placed on an ice rack and stored in a −80 °*C* freezer until analysis. Trophectoderm biopsy was then performed immediately using a non-contact 1.48 *μm* diode laser (RI, Cornwall, UK) with a total of 5–8 trophectoderm cells removed. Prior to blastocoelic fluid aspiration and trophectoderm biopsy, the blastocyst was removed from the embryoscope slide dish and approximately 15–20 *μl* of spent culture media was collected using a sterile individually wrapped 20 *μl* Gilson pipette. The collected media were expelled into a 0.2 *ml* Eppendorf tube and immediately placed on a cold rack and stored at −80°*C* until further analysis. The blastocyst was vitrified post biopsy using Cook blastocyst vitrification media (Cook, Sydney).

All three samples containing DNA and a negative control were sent to our genetic provider, Reprogenetics UK, where genetic analysis was performed using next generation sequencing (NGS). The blastocoel fluid sample and 10 *μl* of the spent media sample were each subjected to a modified version of MDA (Qiagen, UK) using a protocol developed at Reprogenetics specifically for the purpose of amplifying DNA from blastocoel fluid and spent media samples (Babariya and Wells, unpublished). The amplified products were run on a 1% (*w/v*) agarose gel to check for amplification success. Thereafter, Nextera DNA kit (Illumina, UK) was used to prepare libraries from successfully amplified samples followed by 75 *bp* paired end sequencing on a MiSeq (Illumina). Unfortunately, the blastocoel fluid sample resulted in suboptimal amplification and was not subjected to NGS. The sequencing data was analyzed using an in-house custom algorithm to determine the chromosomal copy number.

The trophectoderm biopsy results were euploid for both the embryos. Analysis of cell-free DNA in the corresponding embryo culture media yielded concordant results, indicating that both of the embryos were euploid. The patient had a 3D saline infusion sonohysterography on day 12 of the cycle, which revealed a normal uterine cavity. She was advised to take Primolut^®^ (Bayer Schering Pharma, Germany) 5 *mg* twice daily from day 14 of the cycle until day 25 with Buserelin® (Sanofi-Aventis, Germany) from day 21 as per manufacturer’s instructions. Buserelin was stopped once luteal phase support was commenced. She had a baseline scan on day-1–3 to assess the endometrium and ovaries. Progynova^®^ (Bayer, UK) 2 *mg* three times daily orally and 2 *mg* twice daily vaginally was prescribed on day-3 of the withdrawal bleed. She was advised to have a repeat transvaginal scan 12 days later to assess endometrial thickness. Once the endometrium was >7 *mm* in thickness and had a triple line on ultrasound, luteal support was commenced and embryo transfer was booked 6 days later. Luteal support for cryopreserved embryo transfer cycles included Utrogestan^®^ (Besins, Belgium) 200 *mg* three times daily orally, intramuscular Lentogest (Institut Biochemique SA, Switzerland) three times per week and Crinone^®^ 8% (Merck Serono, UK) twice a day per vaginam. These were continued until 10 weeks of pregnancy.

The suitable blastocyst was warmed on the day of embryo transfer using Cook Warming Kit (Cook, Sidney). The patient was instructed to perform a urinary pregnancy test 16 days later. As the urinary HCG test was positive, the patient was advised to come to the unit for serum HCG. Her serum hCG was 1312.0 *IU/l*. Subsequently, scans at 6 weeks and 8 weeks of gestation confirmed a viable intrauterine singleton pregnancy with normal foetal development. At the time of the write up of the case report, the patient was 20 weeks without any reported foetal or obstetric concerns.

### Ethical Consideration:

This study was approved by the IRAS 214276. This report is based on a finding of the ongoing prospective study.

## Discussion

This is the first UK case report where non-invasive PGT-A screening has been applied in a clinical setting. The cycle resulted in a viable ongoing pregnancy without a reported risk of aneuploidy using serum screening and ultrasound. Our case report suggests that it is straightforward, from a logistical and technical perspective, to incorporate the sampling of blastocoel fluid and spent culture medium into routine clinical practice. While our report cannot provide firm conclusions concerning the efficiency or accuracy of testing based upon sources of cell-free DNA, it was the case that results from spent culture media were concordant with those obtained from trophectoderm biopsy for both embryos tested. This suggests that embryo culture medium may indeed provide source of cell-free DNA suitable for non-invasive PGT-A (niPGT-A). In this case study, blastocoel fluid failed to yield DNA suitable for subsequent analysis. This mirrors our wider experience with this source of cell-free DNA and suggests that the blastocoel may be a less reliable source of embryonic DNA for niPGT-A than spent media. However, the technique of blastocentesis appears to be relatively safe and no evidence of an adverse impact was seen upon embryo development following the procedure in this case.

Invasive trophectoderm biopsy whether for aneuploidy screening or for testing of monogenic disorders or chromosomal rearrangements remains the gold standard method to obtain embryonic DNA (8). Even though an international consensus does not yet exist regarding the clinical practice of PGT-A, laboratory protocols have continued to evolve and are now highly optimized in order to yield a genetic result. In this case report, our patient had two chromosomally normal embryos suitable for transfer, despite the fact that the euploidy rate for individual blastocysts produced by a patient in this age group is typically <20%.

Our case report provides support for the notion that cell-free DNA in spent culture media could be potentially used for non-invasive PGT-A, either to supplement results obtained using embryo biopsy or to entirely replace the biopsy procedure. Clearly, large multicenter studies will be required to clarify the value of niPGT-A strategies such as the one described in this paper. An ongoing preliminary study at our clinic shows an amplification rate of over 65% for spent culture media and trophectoderm with good concordance for euploid embryos (Unpublished data). Release of cell free DNA occurs in human embryo when cultured in vitro via apoptosis and perhaps also *via* other mechanisms (17). Kuznyetsov et al. (2018) recently analyzed cell free DNA from fresh cultured embryos (24–48 *hr*), which showed 100% amplification rate and 98.2% concordance with trophectoderm cells. However, the mean amount of DNA obtained from the embryo culture media in fresh embryos was lower compared to vitrified-warmed blastocysts in their study.

In contrast to the cell-free DNA in embryo culture medium, the results from blastocentesis obtained during the current case were inferior to some other published reports (12, 18, 21). This poor performance is in accord with our wider experience when attempting to harvest and analyze blastocoel DNA, in which only 25% of samples have successfully been amplified. This provides some evidence, albeit from a limited study, that genetic material contained within the blastocoel cavity may be more challenging to utilize in a clinical context than other sources of cell-free DNA. While some studies have reported DNA amplification success rates ranging from 72–96.4% for blastocoel fluid and a high degree of diagnostic concordance for ploidy status (12, 18, 21), it appears that optimal results require highly expanded blastocysts, which were not always available in the current study. Additionally, the lack of amplification and concordance that we have observed using this material may be related to the need to store the sample and then ship to a reference laboratory, potentially allowing further degradation of the DNA. It is also worth noting that, in comparison to the collection of spent culture medium, blastocentesis is a relatively invasive procedure, requiring greater skill, more of the embryologist’s time and a greater exposure of the embryo to suboptimal environmental conditions outside the incubator.

## Conclusion

Trophectoderm biopsy has been instrumental in improving the accuracy rates and diagnostic efficiencies of PGT-A and PGT-M. However, the technique is highly skilled, time consuming, and it necessitates the purchase and maintenance of a laser, and carries with it a small risk of embryonic damage (although minimized in experienced hands). In addition, a limitation to the accuracy of PGT-A following trophectoderm biopsy is chromosomal mosaicism, which poses significant risks for misdiagnosis, as there may be cases where trophectoderm cells do not reflect the chromosomal status of the entire embryo (2). These are some of the combined factors driving the need for alternative, preferably non-invasive, PGT-A methods. The exact origin of cell-free DNA found in spent culture media or blastocoel fluid is currently unknown, but if this is shown to predominantly originate from the ICM, then blastocoel fluid might prove to be an even more appropriate source of DNA for chromosomal analysis than trophectoderm biopsy samples, providing a superior representation of the fetal genetic status. Like any new technology in reproductive medicine, it is paramount to understand the clinical implications of applying the technique in a clinical setting. The experience of this study suggests that removal of blastocoel fluid, sampling of spent culture media, and trophectoderm biopsy can be carried out in parallel. It is possible that gathering genetic information from two or three independent samples of embryo DNA might provide benefits in terms of enhanced diagnostic accuracy, potentially clarifying the cytogenetic status of mosaic embryos. In the future, if appropriately validated and proven to be accurate, niPGT-A methods might even replace trophectoderm biopsy, reducing costs and risks of aneuploidy screening.
